# Spectrum splitting for fast polarization switching of undulator radiation

**DOI:** 10.1107/S1600577516004604

**Published:** 2016-04-19

**Authors:** Ryota Kinjo, Takashi Tanaka

**Affiliations:** a RIKEN SPring-8 Center, 1-1-1 Kouto, Sayo-cho, Sayo-gun, Hyogo, Japan

**Keywords:** undulator radiation, fast polarization switching, spectrum-splitting scheme

## Abstract

A simple scheme to quickly switch the polarity of circular radiation is proposed, which is based on spectrum splitting of undulator radiation. In this scheme, two helical undulators with opposite helicities are placed tandem in one straight section, both of which are divided into several segments. The optical phases between segments are tuned so that light waves from one of the two undulators are out of phase, while those from the other are in phase. Then the radiation spectrum of the former is split and the intensity at the fundamental photon energy vanishes. As a consequence, the monochromated photon beam at the fundamental energy is circularly polarized with the helicity specified by the in-phase undulator, which can be quickly flipped by tuning the optical phase. Numerical calculations carried out to demonstrate the feasibility of the proposed scheme show that a relatively high degree of circular polarization is expected if the angular acceptance of the beamline is not too large.

## Abstract 2

The optical phases between undulator segments are tuned so that selected polarized light from an in-phase undulator survives through the monochromator.

## 1. Introduction   

X-ray magnetic circular dichroism (XMCD), which refers to a difference in the response of a material to circularly polarized light with opposite helicities, is useful for investigating magnetic properties of materials with magnetic elements. It should be noted, however, that the XMCD signal is usually small, and thus the polarization polarity should be flipped as quickly as possible, in order to improve the signal-to-noise ratio in XMCD for materials having impurities. In hard X-ray regions above 5 keV, a diamond crystal phase retarder can be used to generate circularly polarized X-rays, whose polarity is flipped by a small angle change around the Bragg angle of the crystal. Thanks to its simple mechanism, the switching speed with this method typically reaches as high as 100 Hz (Hirano *et al.*, 1992 ▸). Note that a much higher switching speed of 2 kHz has also been achieved (Suzuki *et al.*, 2003 ▸).

The above method to switch the polarization state is not available in the soft X-ray region, where no suitable phase retarders covering the wide range of spectrum and having a high conversion efficiency have been developed. It is thus important that the light source has the ability to switch the helicity of circular polarization quickly. The most straightforward way to switch the helicity is to use an electromagnet helical undulator and flip its polarity. However, it is obvious that the switching speed is significantly limited by the capacity of the power supply and a large closed-orbit distortion probably induced during the polarity flip.

In SPring-8, a helicity switching system, which can in principle be applied to any wavelength regions, has been developed and implemented in the soft X-ray beamlines BL23SU and BL25SU (Shirasawa *et al.*, 2003 ▸). This is based on alternate selection of either of the two photon beams from two helical undulators, which are placed tandem in the same straight but have the opposite helicities. The selection is made by the bump orbit created by five fast kicker magnets installed around the undulators. Although this system has worked for many years in SPring-8 as an indispensable tool for the XMCD applications, there are technical limitations. First, the orbit bump should be relatively large to separate the two photon beams, and should not affect the orbit stability of the electron beam over the storage ring. This means that the closed-orbit distortions induced by the kicker magnets should be within some criteria, which effectively determines the upper limit of the available switching speed. Second, the effective length available for undulators can be significantly reduced because of the kicker magnets. This will be more critical for SPring-8-II, the upgrade project of SPring-8, in which the length of the straight section is supposed to be reduced by nearly 1.5 m.

In this paper, we propose another solution for polarization switching which is hereinafter referred to as the spectrum-splitting (SS) scheme. In the scheme, phase shifters are used instead of kickers to control the polarization state. Because the orbit bump created by the phase shifter to switch the helicity is much smaller than that required in the kicker method, the electron orbit distortion is much smaller, and thus a much higher switching speed would be expected. In the following sections, the principle of the SS scheme is described, together with the results of calculations performed to examine its performance.

## 2. Principle of polarization switching by spectrum splitting   

### 2.1. Spectrum splitting of undulator radiation   

Let us recall the spectrum of the undulator radiation. If we assume that the light generated in an *N*-period undulator is an *N*-cycle sinusoidal wave, the electric field is written as
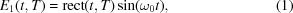
with
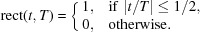
Here, the wave has the fundamental frequency 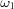 and the time duration *T* which depend on the observation angle as 
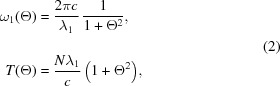
with 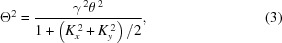
where γ is the Lorentz factor of the electron, θ is the observation angle, and 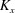 and 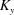 are the horizontal and vertical deflection parameters, respectively.

The Fourier-transformed spectrum of equation (1) in the frequency domain is 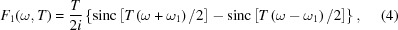
with 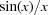 being the so-called sinc function. This gives the well known form to describe the power spectrum of undulator radiation having its peak at 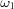: 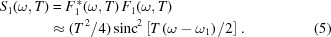
Now let us assume two identical waves having the time duration of 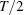 and the time spacing δ in between; the power spectrum is written as 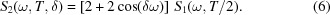
We find that 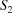 vanishes at 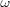 = 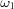 if the condition 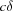 = 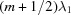 (*m* is an integer) is satisfied.

In a similar fashion, the power spectrum of three identical waves having the time duration of 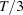 and the time spacing of δ can be written as 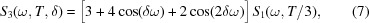
which vanishes at 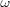 = 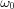 if the condition 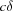 = 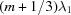 or 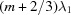 (*m* is integer) is satisfied.

**Figure 1:**
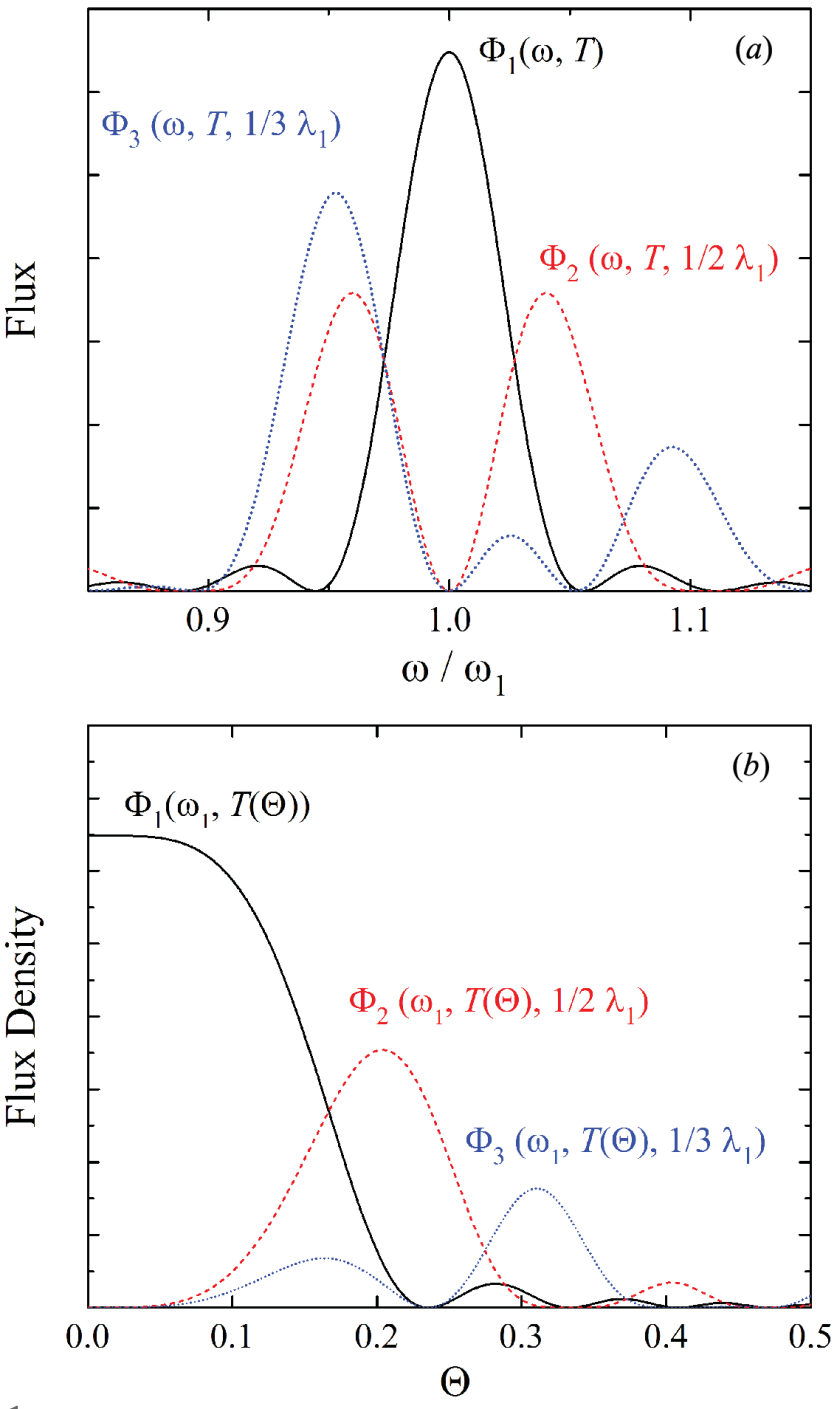
Flux spectrum (*a*) and angular profile (*b*) of the single, double and triple waves with the adjusted time spacings.

Fig. 1 ▸ shows the spectrum of normalized photon flux observed on-axis (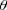 = 0), which is defined by 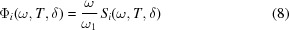
for the single, double and triple waves with the time spacing satisfying the conditions mentioned above. As expected, the photon flux at 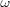 = 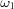 vanishes.

It is worth noting that the photons at the fundamental energy do not actually vanish under the above conditions, but are spatially diffused. This is apparent from Fig. 1(*b*) ▸, which shows angular profiles of Φ. Here, the observation frequency ω is fixed at 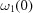.

In general, the above discussion can be extended to arbitrary serial waves with the electric field 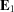 generated by any types of undulators (Tanaka & Kitamura, 2002*a* ▸): 
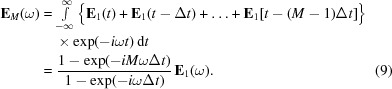
Here, *M* is the number of serial waves corresponding to the number of undulator segments. The power spectrum is given by 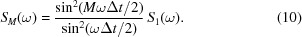
Then, 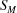 vanishes at the fundamental photon energy if the delay meets the condition 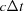 = 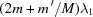 for (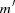 = 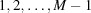).

### 2.2. Polarization switch by the spectrum splitting   

**Figure 2:**
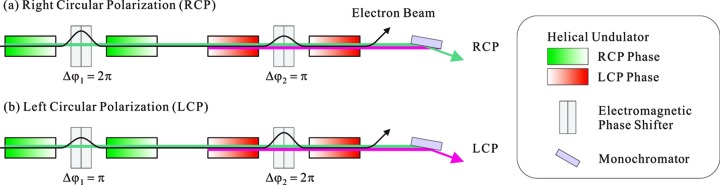
Conceptual drawing of polarization switching using the spectrum-splitting method.

Now let us explain how to switch the helicity of circular polarization by means of the SS scheme described above. Fig. 2 ▸ shows the conceptual drawing of the polarization switching, in which two helical undulators are placed tandem to emit right-handed and left-handed circularly polarized (RCP and LCP, respectively) photons. Each undulator is further divided into two segments with an electromagnetic phase shifter in between. In the operation mode (*a*), RCP components from the two segments are in phase (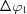 = 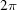), while LCP components have the reverse phase (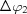 = 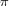). As a result, the RCP photons have a peak at the fundamental photon energy, while the LCP photons vanish at that energy, and thus only the RCP component is brought to the beamline if monochromated at the fundamental energy. In the same manner, only the LCP survives in the operation condition (*b*), in which the electromagnetic phase shifters are tuned so that 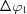 = 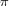 and 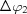 = 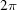.

The polarization switching system based on the SS scheme described above is similar to the conventional kicker system in the point that either of the two polarization components is eliminated. The difference is that the two components are spectrally separated in the former, while they are spatially separated in the latter. Although both systems require electromagnets to create and switch the bump orbits, it is obvious that the typical amplitude of the bump in the former system is much smaller than that in the latter, which significantly relaxes the criteria to correct the trajectory errors induced by the electromagnets, and thus the attainable switching speed would be much higher.

It should be noted, however, that the degree of polarization available in the SS scheme is sensitive to the emittance of the electron beam and angular acceptance of the beamline. This is evident from Fig. 1(*b*) ▸, showing that a larger number of photons with an opposite helicity are found at larger observation angles (Θ). As a result, a larger divergence and acceptance will result in degradation of the degree of polarization.

## 3. Example   

In order to investigate the feasibility of the proposed scheme, we computed expected performances with the electron beam and undulator parameters listed in the Table 1 ▸, which is the typical operation condition planned in SPring-8-II. Note that these parameters are tentative ones as of January 2016, and are subject to change. In the following numerical study, we calculated the undulator field using *RADIA* (Chubar *et al.*, 1998 ▸) and the light properties using *SPECTRA* (Tanaka & Kitamura, 2001 ▸).

**Figure 3:**
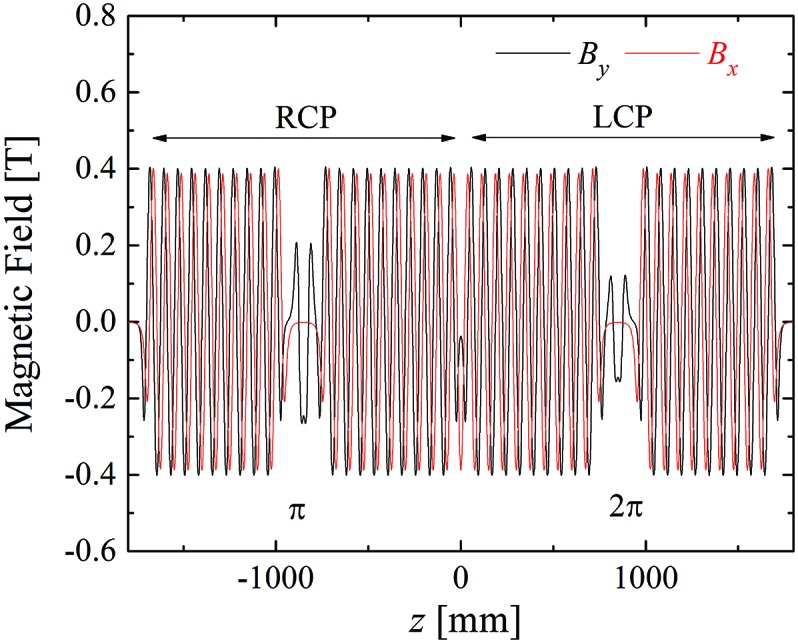
Magnetic field of the system.

We first discuss the design of the helical undulator; in principle, the horizontal and vertical deflection parameters, 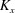 and 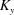, should be equal so that the degree of circular polarization reaches 100%. It is well known that the APPLE-type undulator (Sasaki *et al.*, 1992 ▸) can work as such a ‘perfect helical undulator’ by finely phasing the magnet arrays; however, the phasing motion requires complicated mechanical system and operation. If the experimental users do not need other polarization states than RCP and LCP, we can omit the phasing action to simplify the undulator structure. This concept has been actually adopted in SPring-8 BL23SU, where two fixed-phase helical undulators with opposite helicities have been installed, together with the kicker system, to enable the polarization switching. In the following discussion, we follow this concept; each helical undulator has the fixed phase optimized to satisfy the condition 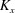 = 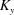 at a specific gap, which is not necessarily the case for other gap values. Fig. 3 ▸ shows the calculated profile of the horizontal and vertical magnetic fields 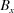 and 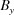 at a gap of 25 mm, in which the dimensions of the magnets are designed to satisfy the condition 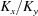 = 1. Note that the upstream helical undulator emits RCP photons, while the downstream one emits LCP photons. Each undulator is divided into two segments with the phase shifter in between, which gives the optical phase of π or 2π at the fundamental energy.

**Figure 4:**
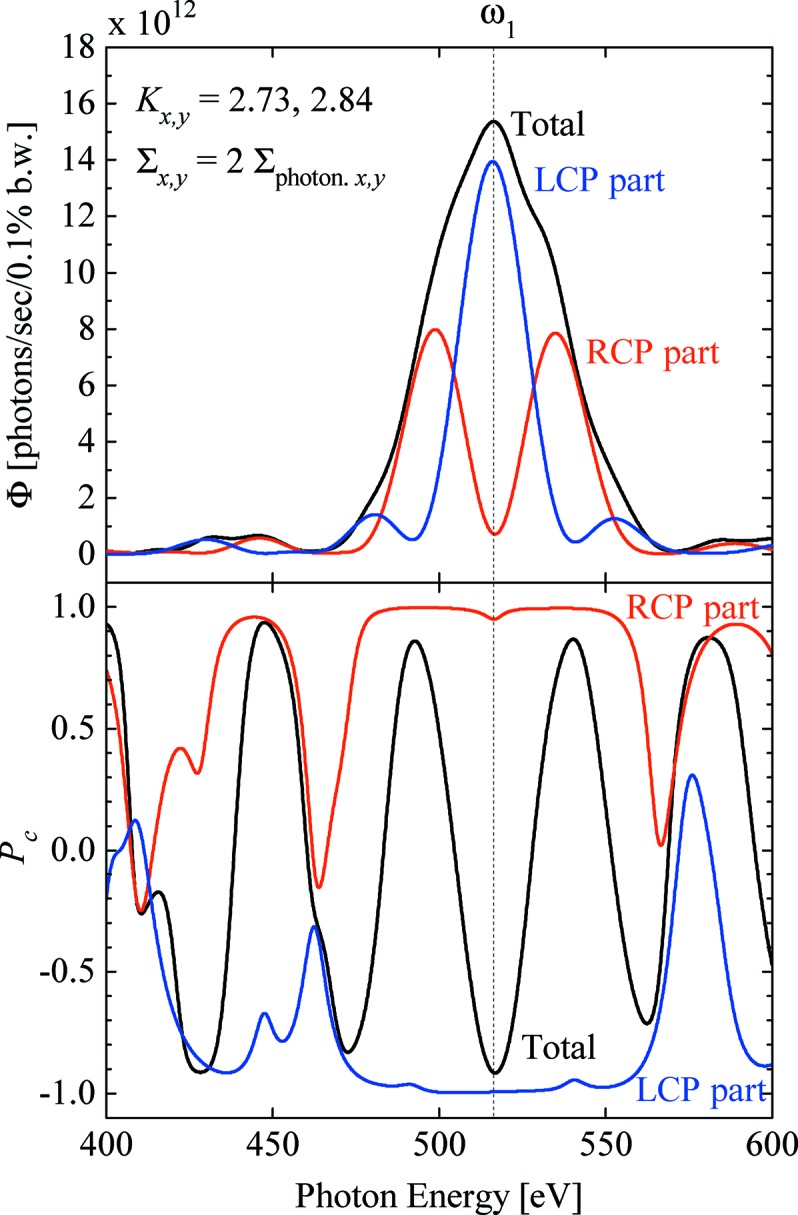
Photon flux (top) and polarization (bottom) from the right polarization (red), left polarization (blue) and all (black) of the system.

Fig. 4 ▸ shows the expected performance in terms of the flux (top) and polarization (bottom) with an assumption that the photons pass through a rectangular slit (front-end slit) of height 0.60 mm and width 0.59 mm located 30 m from the light source (center of the straight section) for the purpose of reducing the heat load on optical elements. The polarization property is given as 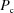 defined by 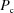 = 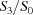, where 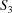 and 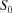 are the Stokes parameters representing the intensity difference between RCP and LCP photons, and the intensity of total photons, respectively. We find that the spectrum of RCP is successfully split; however, the flux at the fundamental energy does not completely vanish. This comes from the finite acceptance angle of the slit and the emittance of the electron beam.

**Figure 5:**
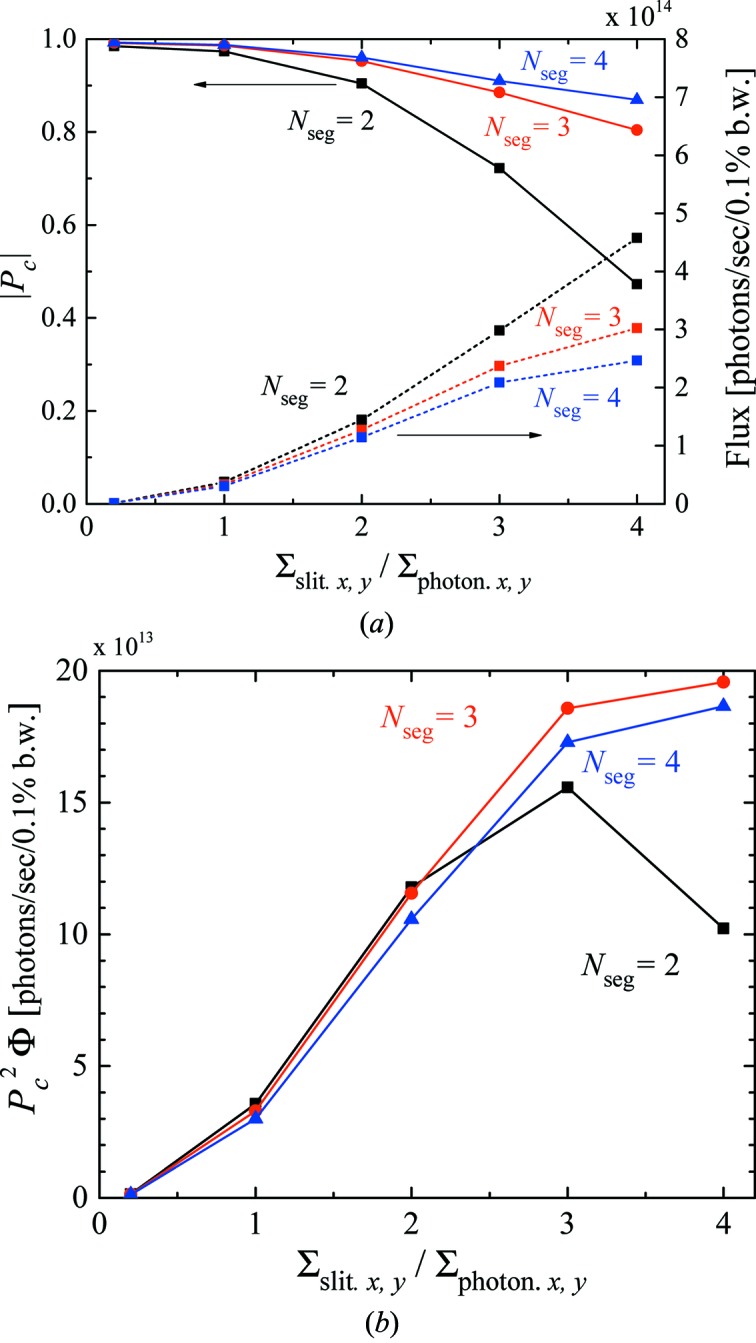
Dependence on the slit aperture for each segmented condition. (*a*) Circular polarization degree. (*b*) Figure of merit.

As mentioned above, the proposed scheme has the difficulty that larger acceptance angles for higher flux will lead to degradation of the degree of polarization. In order to investigate this effect quantitatively, we show the dependence of 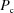 and the flux on the slit aperture for different numbers of segments in Fig. 5(*a*) ▸. Note that, to clarify the discussions below, the interval between the undulator segments is set to zero and the phase shifts are directly included in the radiation calculation. This assumption is made to exclude the effects due to the finite length of the phase shifter, whose design is closely related to boundary conditions such as the required phase shift, available minimum gap, cooling capacity *etc.*, which are out of the scope of this paper. We find that the higher flux is available by applying a larger aperture size, at the expense of degradation of the polarization performances as expected. We emphasize here that the performance can be improved by applying a larger number of segments. For example, 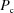 as high as 90% is available even with a relatively large aperture size of 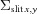 = 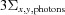, if the number of segments is larger than 3.

In Fig. 5 ▸, we used the averaged 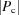 and flux of the photons calculated under two different conditions, in which LCP or RCP photons are dominant. It should be noted that 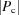 for LCP is around 10% lower than that for RCP. This comes from the difference in angular acceptance of the front-end slit: that for the upstream undulator is slightly narrower than that for the downstream one. However, this problem is somewhat relaxed in practice, and can be further improved, as explained in the following. First, the angular acceptance of light illuminating the sample is not actually defined by the front-end slit, but by another slit for optical systems, which is usually located much further; thus the difference in the angular acceptance may be smaller. Second, the periodic number of the upstream and downstream undulator can be adjusted in the practical design to close their 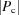 to the average. Third, 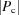 can be reduced by detuning the gap or the phase of the downstream undulator.

Fig. 5(*b*) ▸ shows the the figure of merit defined by 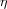 = 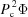, which is generally used to evaluate the signal-to-noise ratio in XMCD measurements. In the view point of the figure of merit, 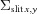 has a peak for 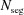 = 2, while it gradually increases with the larger 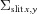 for 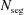 = 3, 4. In other words, the minimum segmentation (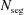 = 2) is enough for the small slit aperture, 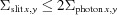.

**Figure 6:**
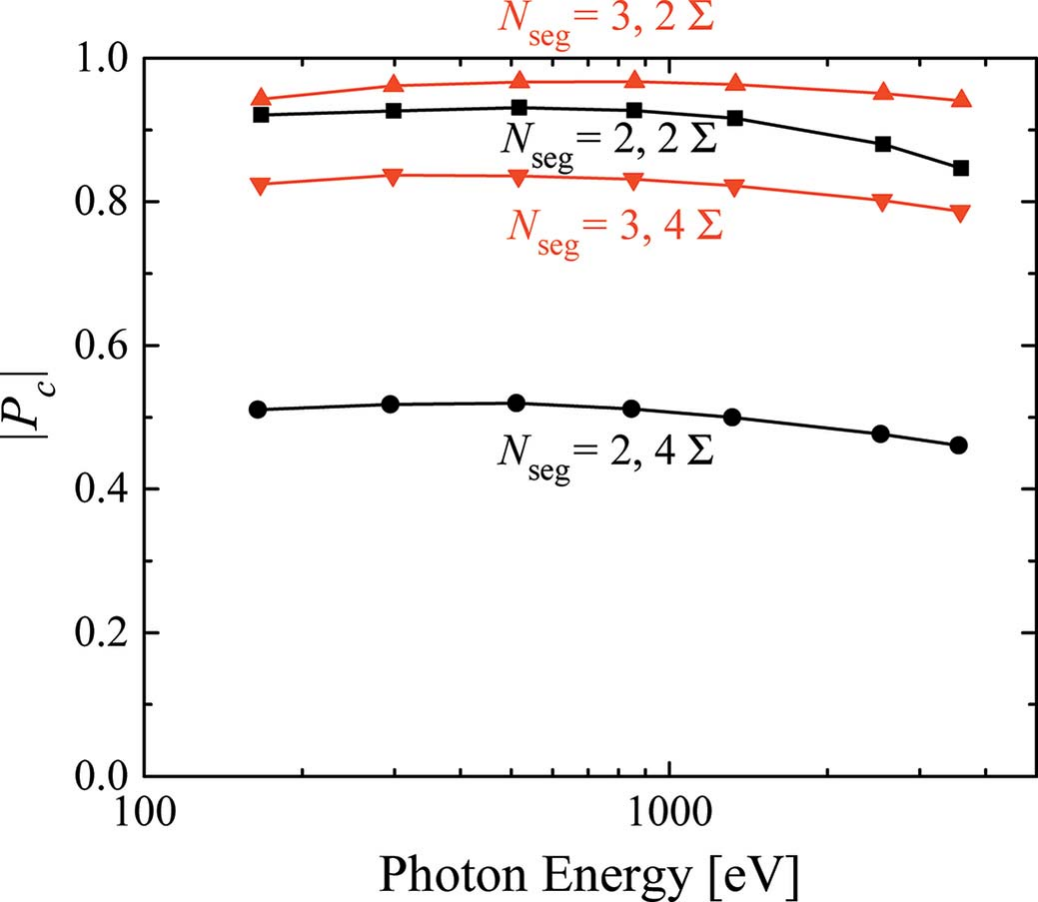
Dependence of the circular polarization degree on the photon energy.

In the discussions above, we have investigated the performance of the SS scheme under a specific condition of 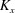 = 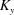 = 2. In practical applications, it is required to change the deflection parameters and fundamental energy. In order to examine the performances at the other energy region of interest, we repeated the above numerical process with 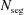 = 2, 3 for different gap values and retrieved the flux and 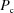 for two different conditions of the slit aperture. The results are shown in Fig. 6 ▸, where 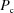 and flux are plotted as a function of the fundamental energy.

In order to carry out accurate XMCD measurements, it is important that 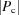 is not only high enough but also keeps constant around the absorption edge. As shown in Fig. 6 ▸, 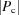 keeps nearly constant over a wide energy range, except a slight decrease in the higher and lower photon energies. The decrease in the higher photon energy comes from the finite emittance of the electron beam, while that in the lower photon energy comes from the difference between 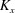 and 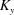, the ratio of which is optimized at the gap of 25 mm (864 eV) by adjusting the magnet dimension of the helical undulator. In any case, 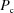 does not show precipitous change and then can be treated as constant during the small energy scan around the absorption edge.

## 4. Comparison with the crossed undulator scheme   

Polarization switching by tuning the optical phase between undulators is also known as the fundamental mechanism of the so-called crossed undulator (CU) (Kim, 1984 ▸) and its extension (Tanaka & Kitamura, 2002*b* ▸, 2003 ▸). They are composed of several undulators emitting vertically and horizontally polarized photons with the phase shifters installed in between. Although the degree of polarization available with the original scheme is not necessarily high under practical conditions, the finite emittance and angular acceptance easily degrade the performance as in the case of the SS scheme with 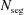 = 2. This difficulty has been solved in the latter scheme, in which the crossed undulator is divided into several segments, which is similar to the SS scheme with 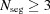.

As mentioned above, the SS scheme has many common points with the CU scheme. It is thus interesting to make a comparison between the schemes. In practice, we have four issues to be discussed: available flux, spectrum purity, characterization of circular polarization, and variation of the source point.

### 4.1. Available flux   

It is obvious that the effective undulator length of the SS scheme is half that of the CU scheme. As a result, it seems that the flux available in the SS scheme is half that of the CU scheme. Although this discussion applies to a specific condition when the deflection parameter is low, we need another discussion when the deflection parameter is high, as in the case of soft X-ray beamlines in SPring-8.

Let 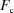 and 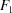 be the photon flux available with the helical and linear undulators, respectively, which have the same specification such as the undulator period. It is easy to show that 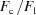 tends to unity for lower deflection parameters, while it approaches 2 for higher deflection parameters (Kincaid, 1977 ▸; Kim, 1986 ▸; Yamamoto & Kitamura, 1987 ▸). As a result, the flux available with the SS scheme is expected to be comparable with that of the CU scheme.

### 4.2. Spectral purity   

It is well known that radiation from a helical undulator does not contain any high harmonic components when observed on axis. This gives us two important advantages over the conventional linear undulator. First, the experimental users do not need to take care of the spurious signal which might be brought by high harmonics that usually survive even after the monochromator. Second, the heat load on optical elements coming from high-energy photons is much lower, which may be more critical in a facility using a high-energy electron beam such as SPring-8. These advantages and disadvantages also apply to the SS and CU schemes, respectively.

### 4.3. Characterization of circular polarization   

In XMCD measurements, evaluating the value of 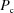 is important for analyzing the experimental data. This is not a complicated task if 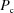 is not sensitive to the experimental conditions, as in the case of the polarization switching with the kicker system. What we need to do is just to compute 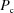 based on the results of the magnetic measurement. In the SS and CU schemes, 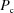 depends on conditions such as the photon energy, electron beam emittance, optical phase between segments, and angular acceptance of the slit. It is thus desirable to directly measure 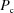 with the same conditions as the target experiments. It should be noted, however, that we need a special setup to accurately measure 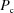, especially in the soft X-ray regions where the availability of phase retarders is limited.

The above problem can be easily solved in the SS scheme; 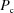 is evaluated by intentionally detuning one of the two helical undulators and measuring the flux from the other (not detuned) undulator. To be specific, let 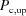 and 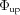 be 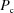 and the flux of the photons by the upstream undulator. Note that the former can be evaluated from the results of magnetic measurement, while the latter can be directly measured, by detuning the downstream undulator and shifting the spectral peak. In the same manner, 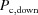 and 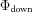 can be also evaluated. Then, 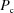 with the actual operation condition, in which both undulators are active, can be determined by 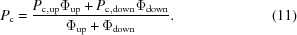



### 4.4. Variation of the source point   

In contrast to the two advantages explained in the former sections, the SS scheme has a disadvantage that two source points exist, *i.e.* the central positions of the two helical undulators. As a result, the source point goes back and forth between the two central positions during the polarization switching. On the contrary, the source point in the original CU scheme is kept constant at the middle point of the straight section. In the segmented CU scheme, the source point also goes back and forth between two points; however, the distances of the source points can be shorter.

**Figure 7:**
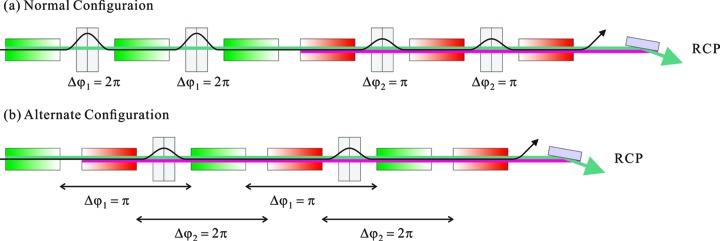
Alternate configuration to reduce the number of the phase shifters and the variation of the source point over each state.

One solution to the above problem is schematically shown in Fig. 7 ▸, where helical undulators with opposite helicities are alternately arranged. This significantly reduces the variation of the source point compared with the original configuration. In addition, the number of phase shifters can be halved with this configuration. It should be noted, however, that we have two drawbacks regarding this configuration. First, the characterization of circular polarization may be more complicated than that mentioned in the previous section. Second, the achievable degree of polarization may be lower, because the larger optical phase between two segments with the same helicity (such as those painted green) works to effectively suppress the spatial diffusion of the photons emitted from the detuned undulator.

## 5. Summary   

We have proposed a new method to realise fast polarization switching based on the SS scheme, which can be applied to any wavelength regions. Because the electron orbit distortion is expected to be much smaller than the conventional kicker method, the switching speed is expected to be much higher. The numerical results show a circular polarization degree of over 90% and a photon flux of over 10^14^ photons s^−1^ (0.1% bandwidth)^−1^ are obtained under practical conditions. We conclude that this scheme is feasible enough to be applied to the fast-polarization switching system for high signal-to-noise XMCD measurements. This scheme may also be adaptable to the so-called afterburner in soft X-ray free-electron lasers, for the purpose of the pulse-to-pulse polarization switching of circular-polarized free-electron lasers.

## Figures and Tables

**Table 1:** Parameters

Electron beam	
Electron energy	6 GeV
Energy spread	9.28 × 10^−4^
Average current	100 mA
Natural emittance	149 pm rad
Coupling	0.1
Undulator section	4.2 m
	
Undulator	
Total length	3.6 m
Period length	75 mm
Horizontal beta value β_*x*_	5.5 m
Vertical beta value β_*y*_	3 m
Deflection parameter *K*_*x*_, *K*_*y*_	3.57, 3.95 @ 15 mm
	2.06, 2.07 @ 25 mm
	0.291, 0.303 @ 60 mm

## References

[bb1] Chubar, O., Elleaume, P. & Chavanne, J. (1998). *J. Synchrotron Rad.* **5**, 481–484. 10.1107/S0909049597013502 15263552

[bb2] Hirano, K., Ishikawa, T., Koreeda, S., Fuchigami, K., Kanzaki, K. & Kikuta, S. (1992). *Jpn. J. Appl. Phys.* **31**, L1209–L1211.

[bb3] Kim, K. J. (1984). *Nucl. Instrum. Methods Phys. Res.* **219**, 425–429.

[bb4] Kim, K. J. (1986). *Proceedings of the 1986 US Particle Accelerator Summer School*, p. 191.

[bb5] Kincaid, B. M. (1977). *J. Appl. Phys.* **48**, 2684–2691.

[bb6] Sasaki, S., Miyata, K. & Takada, T. (1992). *Jpn. J. Appl. Phys.* **31**, L1794–L1796.

[bb7] Shirasawa, K., Hara, T., Takeuchi, M., Hiraya, A. & Kitamura, H. (2003). *Proceedings of the 8th International Conference on Synchrotron Radiation Instrumentation (SRI2003)*, p. 191.

[bb8] Suzuki, M., Kawamura, N. & Ishikawa, T. (2003). *Rev. Sci. Instrum.* **74**, 19.

[bb9] Tanaka, T. & Kitamura, H. (2001). *J. Synchrotron Rad.* **8**, 1221–1228. 10.1107/s090904950101425x 11679776

[bb11] Tanaka, T. & Kitamura, H. (2002*a*). *J. Synchrotron Rad.* **9**, 266–269. 10.1107/s0909049502005472 12091738

[bb10] Tanaka, T. & Kitamura, H. (2002*b*). *Nucl. Instrum. Methods Phys. Res. A*, **490**, 583–591.

[bb12] Tanaka, T. & Kitamura, H. (2003). *Proceedings of the 8th International Conference on Synchrotron Radiation Instrumentation (SRI2003)*, p. 231.

[bb13] Yamamoto, S. & Kitamura, H. (1987). *Jpn. J. Appl. Phys.* **26**, L1613–L1615.

